# Differences in compliance with Surviving Sepsis Campaign recommendations according to hospital entrance time: day versus night

**DOI:** 10.1186/cc12689

**Published:** 2013-04-23

**Authors:** Mónica Almeida, Orquídea Ribeiro, Irene Aragão, Altamiro Costa-Pereira, Teresa Cardoso

**Affiliations:** 1 Mónica Almeida, Unidade de Cuidados Intensivos Polivalente - Hospital de Santo António, Oporto Medical Center, University of Porto, Largo Prof. Abel Salazar, 4099-001 Porto, Portugal; 2 Orquídea Ribeiro, Department of Health Information and Decision Sciences, Center for Research in Health Technologies and Information Systems (CINTESIS), Faculty of Medicine, University of Porto, Alameda Prof. Hernâni Monteiro, 4200-319 Porto, Portugal; 3 Irene Aragão,Unidade de Cuidados Intensivos Polivalente - Hospital de Santo António, Oporto Medical Center, University of Porto, Largo Prof. Abel Salazar, 4099-001 Porto, Portugal; 4 Altamiro Costa-Pereira, Department of Health Information and Decision Sciences, Center for Research in Health Technologies and Information Systems (CINTESIS), Faculty of Medicine, University of Porto, Alameda Prof. Hernâni Monteiro, 4200-319 Porto, Portugal; 5 Teresa Cardoso, Unidade de Cuidados Intensivos Polivalente - Hospital de Santo António, Oporto Medical Center, University of Porto, Largo Prof. Abel Salazar, 4099-001 Porto, Portugal

**Keywords:** Surviving Sepsis Campaign (SSC), 6-hour bundle, compliance rate, hospital mortality

## Abstract

**Introduction:**

Higher compliance with Surviving Sepsis Campaign (SSC) recommendations has been associated with lower mortality. The authors evaluate differences in compliance with SSC 6-hour bundle according to hospital entrance time (day *versus *night) and its impact on hospital mortality.

**Methods:**

Prospective cohort study of all patients with community-acquired severe sepsis admitted to the intensive care unit of a large university tertiary care hospital, over 3.5 years with a follow-up until hospital discharge. Time to compliance with each recommendation of the SSC 6-hour bundle was calculated according to hospital entrance period: day (08:30 to 20:30) *versus *night (20:30 to 08:30). For the same periods, clinical staff composition and the number of patients attending the emergency department (ED) was also recorded.

**Results:**

In this period 300 consecutive patients were included. Compliance rate was (night *vs*. day): serum lactate measurement 57% *vs*. 49% (*P *= 0.171), blood cultures drawn 59% *vs*. 37% (*P *< 0.001), antibiotics administration in the first 3 hours 33% *vs*. 18% (*P *= 0.003), central venous pressure >8 mmHg 45% *vs*. 29% (*P *= 0.021), and central venous oxygen saturation (SvcO_2_) >70%, 7% *vs*. 2% (*P *= 0.082); fluids were administered in all patients with hypotension in both periods and vasopressors were administered in patients with hypotension not responsive to fluids in 100% *vs*. 99%. Time to get specific actions done was also different (night *vs*. day): serum lactate measurement (4.5 *vs*. 7 h, *P *= 0.018), blood cultures drawn (4 *vs*. 8 h, *P *< 0.001), antibiotic administration (5 vs. 8 h, *P *< 0.001), central venous pressure (8 *vs*. 11 h, *P *= 0.01), and SvcO_2 _monitoring (2.5 *vs*. 11 h, *P *= 0.222). The composition of the nursing team was the same around the clock; the medical team was reduced at night with a higher proportion of less differentiated doctors. The number of patients attending the Emergency Department was lower overnight. Hospital mortality rate was 34% in patients entering in the night period *vs*. 40% in those entering during the day (*P *= 0.281).

**Conclusion:**

Compliance with SSC recommendations was higher at night. A possible explanation might be the increased nurse to patient ratio in that period. Adjustment of the clinical team composition to the patients' demand is needed to increase compliance and improve prognosis.

## Introduction

Severe sepsis is associated with high morbidity and mortality [1]. It is one of the major causes of death worldwide with an associated mortality of 29%, causing as many deaths annually as those from acute myocardial infarction [2].

It is expected to become even more prevalent due to the aging population, the increasing number of immunocompromised patients, and the increasing resistance of bacteria to antimicrobial therapy. If not treated promptly, it leads to multiple organ failure and death [3].

The vast majority of sepsis patients are initially managed outside the intensive care unit (ICU) environment, by different medical teams, creating the need to standardize practices; this led to the development of the 'Surviving Sepsis Campaign' (SSC), that included the publication of a summary of the existing evidence on severe sepsis treatment and aimed to standardize clinical practice, improve standards of care, and reduce mortality [4].

It has gone through a process of 'bundle' definition; a bundle is a group of interventions related to a disease process, that when executed together produce better outcomes than when implemented individually [5].

It was organized around two time frames: the resuscitation care bundle to be accomplished in the first 6 h of the diagnosis and the management care bundle for the next 24 h [6], mainly reserved for the intensive care environment.

The authors evaluate differences in compliance with the Surviving Sepsis Campaign 6-h bundle according to hospital entrance time (day *vs*. night), in patients with community-acquired severe sepsis and its impact on hospital mortality.

## Materials and methods

### Study design and patient population

Prospective cohort study including all consecutive adult patients admitted to the ICU, of a 600-bed university, tertiary-care hospital from 1 December 2004 until 31 May 2008.

The inclusion criteria were diagnosis of community-acquired severe sepsis (CASS) in the emergency department (ED) and posterior admission to the ICU. All patients completed the follow-up until hospital discharge.

This study was approved by the Institutional Review Board of Hospital de Santo António, Oporto Hospital Centre, and informed consent was waived due to the observational nature of the study.

### Definitions

Community-acquired infection was defined as the onset of infection before hospital admission or not present at admission becoming evident in the first 48 h [7]. Sepsis and sepsis-related conditions were defined according to the criteria proposed by the American College of Chest Physicians/Society of Critical Care Medicine [8].

For the analysis of compliance with the 6-h SSC bundle [9], only patients with severe sepsis criteria on ICU admission were included in the study. Time zero was defined as hospital entrance time, that is, the time when the patient arrived at the hospital (administrative registration time, before Emergency Department (ED) triage).

### Data collection and management

Data collected included demographic and clinical characteristics of patients: age, gender, severity of sepsis, SAPS II score in the first ICU day, ICU and hospital length of stay, and ICU and hospital-mortality.

Compliance rate with each component of the 6-h bundle was also recorded, namely: serum lactate measurement, fluid-resuscitation, blood or others specimens drawn for appropriated cultures, antibiotic administration, achievement of central venous pressure (CVP) >8 mmHg, and central venous oxygen saturation (SvcO2) >70%.

The study population was stratified in two groups according to hospital entrance time: day (08:30 to 20:30) *vs*. night (20:30 to 08:30). Clinical staff composition in the ED was also recorded along with the number of patients attending the department in each period.

### Statistical analysis

Data were described with means and standard deviations for continuous variables, or with medians and interquartile ranges if data displayed a skewed distribution. Comparisons were performed using Pearson χ^2 ^or Fisher's exact test for categorical variables and Mann-Whitney test for continuous variables.

The risk factors studied for association with hospital mortality included age, sex, SAPS II, severity of sepsis, hospital entrance time, and full compliance with the 6-h bundle. Variables associated with hospital mortality in the univariate analysis (*P *value < 0.1) and with a clear relationship described previously in the literature were selected for the multivariable analysis. The results of the multivariable models are expressed as odds ratio (OR) with 95% confidence interval (CI_95%_) and *P *values. The calibration was tested using the Hosmer-Lemeshow goodness-of-fit test. The significance level was defined as *P *< 0.05.

Data were analyzed using SPSS, version 19 for Windows (Chicago, IL, USA).

## Results

During the study period, 1,223 patients were admitted in the ICU; of those 300 (25%) had CASS: 123 (41%) entered the hospital during day and 177 (59%) overnight. There were no significant differences regarding age, sex, SAPS II, ICU or hospital length of stay, and ICU or hospital mortality rate between both periods (Table 1).

**Table 1:** Demographic and clinical characteristics of all patients admitted in the ICU, patients with CASS, comparing day *vs*. night patients.

Patients' characteristics	**Total ICU patients**, *n *= 1223	**ICU patients with CASS**, *n *= 300	**Day patients**, *n *= 123	**Night patients**, *n *= 177	Day *vs*. night, *P *value
Age (years), mean (SD)	55 (19)	58 (17)	60 (17)	57 (17)	0.164^a^
Male sex, n (%)	799 (65)	178 (59)	94 (56)	84 (64)	0.137^b^
SAPS II, mean ± SD	45 ± 16	58 ± 17	48 (15)	47 (17)	0.506^a^
ICU LOS, median (IQR)	7 (3-13)	8 (3-15)	8 (3-16)	8 (3-13)	0.587^c^
ICU mortality rate, n (%)	357 (29)	100 (33)	60 (36)	40 (31)	0.365^b^
Hospital LOS, median (IQR)	16 (7-32)	16 (7-27)	17 (7-31)	13 (6-24)	0.106^c^
Hospital mortality rate, n (%)	413 (34)	111 (37)	67 (40)	44 (34)	0.281^b^

^a^Independent sample t-test.

^b^Chi-square test.

^c^Mann-Whitney test.

CASS: Community-acquired severe sepsis; ICU: Intensive care unit; IQR: Interquartile range; LOS: Length of stay; SD: Standard deviation.

Overall compliance with the SSC 6-h bundle was: 52% for serum lactate measurement, 47% for blood cultures drawn, nearly all patients had fluid administration if hypotension was present as well as vasopressor administration if not responsive to fluid challenge, 25% had antibiotic (ATB) administered in the first 3 h, 36% achieve a CVP >8 mmHg and 4% a SvcO_2 _>70% in the first 6 h of hospital entrance time. Compliance rate with the entire 6-h bundle was 2%. If CVP and SvcO_2 _are left aside (that is, considering only actions 1 to 5 of Table 2), compliance rate increased to 12%.

**Table 2:** Comparison of compliance with each component of the 6-h bundle, according to hospital entrance time.

Components of the 6-h bundle	Total, *n *(%)	Day, *n *(%) (08:30 to 20:30)	Night, *n *(%) (20:30 to 08:30)	*P *value
1 - Serum lactate measurement	156 (52)	82 (49)	74 (57)	0.171^a^
2 - Blood cultures drawn	140 (47)	63 (37)	77 (59)	<0.001^a^
3 - Antibiotic administration within the first 3 h of hospital entrance time	70 (25)	28 (18)	42 (33)	0.003^a^
4 - Fluids administration in patients with hypotension	200 (100)	107 (100)	93 (100)	-
5 - Administration of vasopressors when indicated	197 (100)	107 (99)	90 (100)	-
6 - CVP >8 mmHg	75 (36)	33 (29)	42 (45)	0.021^a^
7 - SvcO2 >70%	9 (4)	2 (2)	7 (7)	0.082^b^

Compliance with 6-h bundle, *n *(%)	5 (2)	1 (1)	4 (3)	0.172^a^

^a^Chi-square test.

^b^Fisher exact test.

CVP: Central venous pressure; SvcO2: Central venous oxygen saturation.

Compliance with each component of the bundle was significantly different according to hospital entrance time (night *vs*. day): blood cultures drawn (59% *vs*. 37%, *P *< 0.001), antibiotic administration in the first 3 h of hospital entrance (33% *vs*. 18%, *P *= 0.003), and achievement of a CVP >8 mmHg (45% *vs*. 29%, *P *= 0.021) (Table 2).

Time to get specific actions (night *vs*. day): serum lactate measurement (4.5 *vs*. 7 h, *P *= 0.018), blood cultures drawn (4 *vs*. 8 h, *P *< 0.001), ATB administration (5 *vs*. 8 h, *P *< 0.001), central venous pressure (8 *vs*. 11 h, *P *= 0.01), and SvcO_2 _monitoring (2.5 *vs*. 11 h, *P *= 0.222) was also lower during the night period (Table 3).

**Table 3:** Comparison between night and day periods of time to achieve each component of the 6-h bundle (hours)

6-h bundle component	Day, h	Night, h	*P *value
Serum lactate measurement, median (IQR)	6:97 (1:27-25:18)	4:48 (0:83-14:02)	0.018*
Blood cultures drawn, median (IQR)	8:17 (2:74-25:54)	4:06 (1:55-10:83)	<0.001*
Antibiotic administration, median (IQR)	8:18 (4:25-16:17)	4:95 (2:02-9:89)	<0.001*
CVP >8 mmHg, median (IQR)	10:94 (5:17-22:50)	7:83 (1:88-17:37)	0.010*
SvcO_2 _>70%, median (IQR)	10:65 (8:78-22:17)	2:47 (1:02-29:64)	0.222*

^a^Mann-Whitney test.

CVP: Central venous pressure; IQR: Interquartile range; SvcO2: Central venous oxygen saturation.

Regarding the ED clinical team: the nursing team had a similar composition in number and experience, distributed in three shifts (00:00-08:00, 08:00-16:00, 16:00-00:00); the medical team had few elements at night with a higher proportion of less differentiated doctors, mainly between 00:00-08:30.

During the study period 516,619 patients were attended in the ED: 49,901 (10%) between 00:00 and 08:00, with a mean time to first medical observation of 62 min; 277,587 (54%) between 08:00 and 16:00, with a mean time to first medical observation of 75 min; and 189,131 (36%) between 16:00 and 00:00 with a mean time to first medical observation of 67 min.

Crude hospital mortality rate for patients with severe sepsis was 40% in day patients and 34% in night patients (*P *= 0.281).

Variables significantly and independently associated with hospital mortality were age (adjusted OR = 1.022 per year), SAPS II (adjusted OR = 1.039 per point), and septic shock (adjusted OR = 1.970) (Table 4).

**Table 4:** Selection of variables significantly and independently associated with hospital mortality, using logistic regression.

Variables	Total	Crude OR	*P *value	Adjusted OR	CI_95%_
Age*, mean (SD)	59 (17)	1.031	<0.001	1.022	1.005-1.039
Male sex, *n *(%)	178 (59)	1.360	0.311		
SAPS II, mean (SD)^a^	48 (16)	1.052	<0.001	1.039	1.019-1.059
Septic shock, *n *(%)	197 (66)	3.284	<0.001	1.970	1.077-3.602
Night hospital entrance (20:30-08.30), *n *(%)	131 (44)	0.770	0.282		
Compliance with the 6-h bundle	5 (2)	0.000	0.999		

^a^Increase in the odds ratio per point.

OR: Odds ratio; SAPS: Simplified Acute Physiology Score; SD: Standard deviation.

*Increase in the odds ratio per year.

## Discussion

Patients with CASS attending the hospital overnight had higher compliance rate with each component of the 6-h bundle than those entering during day. Time to get specific actions done was also lower at night.

A possible explanation relies on the lower number of patients entering the ED overnight for the same number of nurses, increasing the nurse to patient ratio. In Portugal, as in many other European countries, nurses are responsible for peripheral venous puncture for blood cultures drawn and medicine administration. Higher availability of the nursing staff might have speed blood cultures drawing, antibiotics administration, and invasive monitoring, suggesting that the number of nurses during the day might not be sufficient to target the needs. Although invasive monitoring is a medical procedure, nurses are in charge of the set-up and monitoring.

Even medical tasks like serum lactate measurement and invasive monitoring were done faster overnight despite the low number of doctors and the higher proportion of less differentiated professionals. Between night and day, the differential time to get specific actions done was >2 h but the differential to first medical observation was only 13 min. Of note is the fact that the mean time to first medical observation was >1 h in all periods, suggesting that the medical team also needs to be reinforced in all shifts in order to decrease the length of time for attending such severe group of patients and improve prognosis.

A national survey performed by Carlbom et al. [10] evaluating which barriers most commonly affect the application of protocol-based sepsis resuscitation in the ED found three top barriers with 'nursing staff required to perform EGDT' as the most frequently ranked; 'monitoring of central venous pressure' and 'identifying septic patients' werethe second and third most common problems, respectively. Our study reinforces the need to adapt clinical team composition to the different patients' demands in the 24-h period.

On the other hand, invasive techniques are more difficult to implement and the attending physicians tend to think that they are too busy for time-consuming invasive procedures, thus devaluing the importance of those actions in management and treatment of septic patients if they are not sensitized for these questions [11].

Nevertheless, previous multicenter studies have shown a significant impact of compliance with the SSC 6-h bundle in reducing mortality from severe sepsis [12-15]. Although crude ICU and hospital mortality rate for patients with severe sepsis were lower during the night, they did not reached statistical significance. Variables independently associated with hospital mortality were age, SAPS II, and septic shock. Surprisingly, compliance with the 6-h bundle was not associated with lower hospital mortality, which might be explained by the low number of patients that had full compliance with the recommendations, not allowing the establishment of a proper correlation.

The current study has the great advantage of being prospective. Clear definitions were used to allow comparisons between studies. Data collection was thorough with all protocols completed and no missing data per item minimizing information bias. All patients completed follow-up until hospital discharge.

Time zero was clearly defined as hospital entrance time, making data more objective. However, selecting hospital entrance time instead of 'sepsis recognition time' may have biased the results towards lower compliance.

In fact, time zero has been the subject of great debate [16]. Some authors [17] consider time zero as the moment when the patient becomes hypotensive or when serum lactate is ≥4 mmol/L, while others consider time zero as the moment of the diagnosis, regardless of how long the patient has been in hospital [18]. The use of such different definitions may markedly affect the assessment of compliance with interventions, making comparisons between studies difficult. Time zero definitions based on the diagnosis time of sepsis, of hypotension, or of high lactate levels are later than the disease onset and may give doctors false reassurance, by falsely increasing the compliance rate.

Overall, compliance with the 6-h bundle was very low (only 2%) and it decreased as more invasive techniques were needed. If invasive monitoring is left aside (considering only actions 1 to 5 of Table 2), the compliance rate would increase to 12%, a rate similar to the results founded in other multicenter studies [19-22] despite the definition of time zero adopted, that overestimates time to get actions done, underestimating overall compliance.

This study also has additional limitations that should be acknowledged. The research was performed in a single institution and the number of patients with CASS was relatively small. It included only patients admitted to the ICU, not evaluating patients treated in intermediate care units.

## Conclusions

There is a clear need to review the clinical team composition, adjusting the number of doctors and nurses in each shift to the patients' needs, to get actions done in a timely manner, and improve prognosis.

Periodic audits of clinical performance, though challenging, are essential to the identification of simple operational problems, that when addressed might improve quality of care and provide feedback for team motivation.

## Key messages

• The SSC 6-h bundle compliance depends on the patients' hospital entrance time.

• Compliance with individual components of the SSC 6-h bundle was higher during the night period.

• The higher and faster compliance during the night period seems to be dependent on the higher nurse to patient ratio.

• Clinical practice could be improved by adjusting the ED clinical team (doctors and nurses) to the patients' demands in each period of the day.

• Periodic audits of clinical performance, though challenging, are essential to identify structural problems, improve quality of care, and to provide feedback for team motivation.

## List of abbreviations

ATB: Antibiotic; CASS: Community-acquired severe sepsis; CI: Confidence interval; CVP: Central venous pressure; ED: Emergency Department; EGDT: Early goal-direct therapy; ICU: Intensive care unit; MAP: Mean arterial pressure; OR: Odds ratio; SAPS II: Simplified Acute Physiological Score; SSC: Surviving Sepsis Campaign.

## Competing interests

The authors declare that they have no competing interests.

## Authors' contributions

All authors have made substantial contribution on the conception, design, and acquisition of data and/or analysis and interpretation of data, as well as in the drafting, revision, and final approval of the version to be published.
